# Dark personality traits and deception, and the short dark tetrad (SD4) as integrity screening instrument

**DOI:** 10.1038/s41598-023-50968-7

**Published:** 2024-01-03

**Authors:** Eric Rassin, Melissa de Roos, Josanne van Dongen

**Affiliations:** Department of Psychology, Education and Child Studies, Erasmus School of Social and Behavioural Sciences, Rotterdam, The Netherlands

**Keywords:** Risk factors, Signs and symptoms

## Abstract

Dark personality traits (Machiavellianism, Narcissism, Psychopathy, and Sadism) have been associated with aversive, unethical, and criminal conduct. Concise measurement tools such as the Short Dark Tetrad (SD4) are popular, because they lend themselves as screening instruments. As such, the scores on these scales are used in various decision-making contexts, and they can have considerable effects on the lives of people who display an unfortunate scoring pattern. The present study explored to what extent high SD4 scores are actually predictive of deceptive behaviour in a matrix puzzle task, in a general community sample (*N* = 751). Results indicated that 9.9% of participants lied, that is, exaggerated their performance on the matrix task, hoping to increase their likelihood of financial reward. These cheating participants scored higher on all four dark traits. Nonetheless, the overlap between SD4 distributions made it impossible to determine cut-off scores in an attempt to consider scores as actual predictors of deception proneness. When framed in likelihoods, some scores can be diagnostic of deception proneness. Particularly in the context of statement validity assessment, characterized by tools with modest to poor accuracy, SD4 scores may add to diagnostic accuracy.

## Introduction

In 2014, Jones and Paulhus introduced the Short Dark Triad (SD3), a useful self-report measure that taps Machiavellianism, Narcissism, and Psychopathy^1^. The SD3 aimed to make it possible to study the dark traits simultaneously as separate constructs and in combination. In 2021, the SD3 was updated into the Short Dark Tetrad (SD4) to include Sadism as a fourth related yet distinct dark trait^2^. Despite the inclusion of a fourth trait, the SD4 is, like its predecessor, a concise, short scale that is suitable as screening instrument in personality, social, and organizational settings.

The dark personality traits have been associated with unpleasant, unethical, and even criminal behaviour. For example, the original Cleckley criteria for psychopathy included unreliability, untruthfulness, and insincerity as key indicators^3^. The traits are also associated with psychiatric syndromes. For example, psychopathy has overlap with the antisocial personality disorder. Likewise, Narcissism and Sadism have clinical counterparts^4^.

If the SD4 is to be used as a screening instrument, it is crucial that the scale actually predicts the target behaviour. For example, it would seem unfair to suspect someone of deception proneness (lack of integrity) merely because of their elevated score on the SD4 if it were not certain that such an elevated score actually predicts deception, not just theoretically but also empirically. As to deception propensity, research has indicated that SD3 scores correlate with deception self-report measures^5–7^. Likewise, all four dark traits, as measured with the SD4 are found to correlate with self-reported deception^8^. In a few studies, correlations between dark personality traits and behavioural deception were computed. For example, Machiavellianism was found to correlate with deception in a communication game that participants could win by lying to a fictitious other game player. Further, psychopathy as measured with the SD3 was found to correlate with the magnitude of exaggerating one’s own performance on a matrix task. In that task, participants had to report how many out of 20 matrix puzzles they had successfully solved. Unknown to them, some of the puzzles were unsolvable, so the researchers could easily find out whether participants had exaggerated (lied about) their performance^9^. Finally, in a coin flip game, cheating (i.e., flipping the coin more often than permitted, in order to obtain the desired result) was associated with all three dark traits of the SD3^10^.

In sum, there is some evidence for the suitability of the dark traits as predictors of deceitfulness. Hence, the SD4 can be considered as a screening instrument, not just for the dark traits themselves^2^, but also for deceitfulness. The use of the SD4 in that manner may be of interest for example in the context of statement validity analysis. Note that in that context, assessors commonly rely on verbal analyses such as criteria-based content analysis (CBCA), or the analysis of nonverbal behaviour. Admittedly though, the well documented accuracy of CBCA does not exceed 70%^11^, while that of analyses of nonverbal behaviour gets stuck at approximately chance level (54%)^12^. Hence, any additional information on the truthfulness of a statement is welcome. Obviously, information about the integrity or deceitfulness of the sender may be of interest, although a score on a test cannot provide certainty as to the validity of a statement given by the person who took the test. That said, psychological tests are oftentimes used in this manner.

The purpose of the current research was twofold. First, we sought to correlate the SD4 with a behavioural measure of deceit. To the best of our knowledge, the SD4 has been studied in relation to self-reported deception^8^, but not with a behavioural measure of deceitfulness. Second, we wanted to elaborate on the possibility of employing the SD4 as a screening instrument of deception proneness. As such, pronounced scores on the SD4 may give additional information in the context of, for example, witness statement validity assessment.

## Results

Of the 751 participants, 74 (i.e., 9.9%) reported to have solved more than 13 matrices and were thus classified as liars. There were no gender differences between truth tellers and liars: *χ*^*2*^(3) = 5.0 *p* = 0.172; *BF*_*10*_ = 0.65, but the liars were younger (*M* = 25.25, *SD* = 8.27) than the truth tellers (*M* = 28.26; *SD* = 11.94; *t*{732] = 2.07, *p* = 0.039, *BF*_*10*_ = 0.78). On average, truth tellers reported to have solved 6.76 (*SD* = 3.79) matrices, while liars reported a mean of 16.32 (*SD* = 2.30; *p* < 0.001, *BF*_*10*_ > 1,000).

Table 1 presents the scores on the SD4 of truth tellers and liars, and the results of separate logistic regressions in which lying was predicted with one single dark trait (and age). As can be seen, liars scored consistently higher on the dark traits, and thus, all dark traits predicted deceitfulness, even when applying a Bonferroni correction, setting *p* at 0.01.

**Table 1:** Mean Scores (and Standard Deviations) of Truth Tellers and Liars, and Regression Analyses of Each Dark Trait Predicting Deception (Controlled for Age).

	Mean of truth tellers (*n* = 677)	Mean of liars (*n* = 74)	*r*	*Beta*	*Wald*	*p*
Machiavellianism	21.58 (3.68)	23.20 (3.45)	0.12	0.11	10.04	0.002
Narcissism	19.27 (4.54)	20.73 (4.53)	0.10	0.08	7.73	0.005
Psychopathy	14.20 (4.34)	16.42 (5.28)	0.15	0.10	15.86	< 0.001
Sadism	15.69 (5.00)	18.59 (5.92)	0.16	0.10	16.74	< 0.001

To explore which of the dark traits was most strongly related to cheating on the matrix task, the correlations in Table 1 were tested against each other pairwise. None of the dark traits stood out correlation-wise significantly with *p*s ranging between 0.238 and 0.843. Next, a logistic regression analysis was run in which lying was predicted by the four traits simultaneously, plus age. Again, none of the predictors remained significant with *Betas* of 0.07 (Wald = 3.05; *p* = 0.081) for Machiaveliianism, 0.20 (Wald = 0.41; *p* = 0.524) for Narcissism, 0.52 (Wald = 2.63; *p* = 0.105) for Psychopathy, 0.51 (Wald = 3.15; *p* = 0.076) for Sadism, and -0.02 (Wald = 2.62; *p* = 0.106) for age (Nagelkerke *R*^*2*^ = 0.08).

Finally, correlations were computed between the dark traits and the number of lies, or rather, the magnitude of the lies (that is, the precise self-reported number of solved matrices). Given that there were seven unsolvable matrices, this number could range between one and seven within the subsample of liars (*n* = 74). The analyses indicated that Machiavellianism (*r* = 0.01, *p* = 0.917, *BF*_*10*_ = 0.09) and Sadism (*r* = 0.15, *p* = 0.206, *BF*_*10*_ = 0.20) did not correlate with the extent of the exaggeration of one’s performance, but Narcissism (*r* = 0.31, *p* = 0.008, *BF*_*10*_ = 3.0) and Psychopathy (*r* = 0.34, *p* = 0.003, *BF*_*10*_ = 7.4) did. In a regression analysis in which the number of lies was predicted with Narcissism and Psychopathy, the former did not remain a significant predictor (standardized *β* = 0.20, *p* = 0.112), but Psychopathy did (*β* = 0.25, *p* = 0.038; *R*^*2*^ = 0.15).

## Discussion

The goal of the present study was to further strengthen the association between dark traits and deceitfulness, and to explore the possibility of using the SD4 as a screening tool for integrity or deceitfulness. In our sample of community volunteers, 9.9% exaggerated their performance on a matrix test, thus increasing their perceived likelihood of financial profit. This proportion of liars is relatively small in comparison with the grand mean base rate of 48% of liars in this kind of studies^13^. Our lying participants, scored consistently higher on the dark traits than did their honest peers, and thus SD4 scores predicted deceitfulness significantly. Although there may be reasons to believe that some dark traits are more closely related to deceit than others, our analyses did not allow for such a differentiation. Indeed, all dark traits correlated significantly with each other (*r*s between 0.21 and 0.51, *p*s < 0.001, *BF*_*10*_s > 1000). As to the magnitude of lying, that is, how fiercely did participants overreport their performance, Narcissism and particularly Psychopathy correlated with this outcome. The latter findings confirm previous reports^9^. The observed negative association between deceitfulness and age was not anticipated. Possibly, the older participants were less affected by the possibility of winning 10 euro.

**Figure 1:**
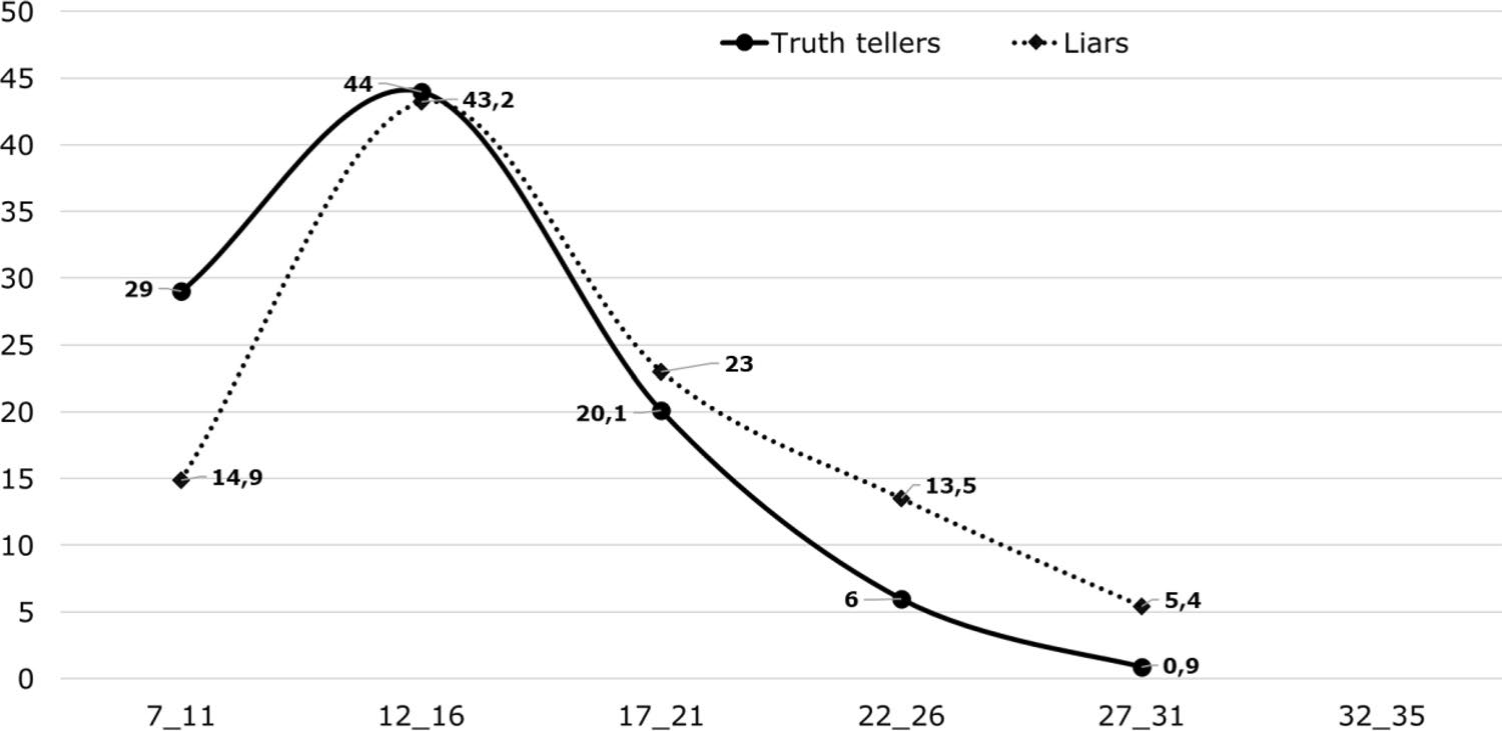
Distribution of SD4 psychopathy scores for truth tellers and liars.

That our findings confirm that dark personality traits are associated with deception propensity, does not automatically make the SD4 a good screening instrument for deception. Note that we are aiming for a screening measure not for the dark traits per se^2^, but for the proneness to lie in a situation where lying is hedonistically beneficial. Our findings do not allow for useful cut-off scores, because all options will suffer a vast sensitivity–specificity trade-off, due to the overlap of the distributions. Given that Psychopathy was a successful predicter of deceitfulness in this and previous studies^14^, we use the distribution of this trait as example. The relative distributions of Psychopathy scores for truth tellers and liars are displayed in Fig. 1 with Psychopathy score ranges on the X-axis, and percentages of participants on the Y-axis. That is, both subsamples were set at 100%. From Fig. 1, it can be concluded that despite the significant association between Psychopathy and deceitfulness, the two Psychopathy distributions of truth tellers and liars strongly overlapped.

However, there is an alternative approach that may make the SD4 more useful in this regard, namely the likelihood-ratio approach. This approach is common in forensic sciences^15^ and was introduced in forensic psychology recently^16^. Applied to our analysis, findings suggest that liars are approximately six times more likely to score in the range of 27 to 31 (5.4%) than are truth tellers (0.9%). This information can be informative in various decision-making contexts. It is important to note that the likelihood ratio of six does not necessarily imply that a score between 27 and 31 makes it six times more likely that the person will lie in a pertinent situation. That would only be so if the base rate of liars can be assumed to be 50%, or in Bayesian terms: If the prior odds are one. That said, when framed in terms of likelihoods, the scores on the SD4 can inform decision makers, for example about the validity of a witness statement. As mentioned, commonly used tool such as CBCA, or the analysis of nonverbal behaviour have very limited diagnosticity^11,12^. Translated to likelihood ratios, the accuracy of CBCA is approximately (70% / 30% =) 2, and that of nonverbal analyses (54%/46% =) 1. Against this background, the likelihood ratio of a pronounced SD4 score would definitely add to the assessment of statement validity, of course in combination with the other approaches, and based on even larger samples than the current.

A methodological limitation of the current findings lies in the matrix task, in that it is not completely sure that participants who reported to have solved more than thirteen matrices actually lied. Alternatively, they might have overestimated their performance, even accidentally. In addition, participants who reported to have solved thirteen matrices or less were classified as truth tellers but might have exaggerated their performance, nonetheless. Further, we created a low stake situation, albeit that there is no reason to argue that dynamics in high stake situations differ^17^. Also, the paradigm dictates that lying participants have a low risk of detection, and we did not include a manipulation check to establish with certainty that participants found out that some matrices were unsolvable, and actually believed that their performance would not be checked. In fact, their performance was indeed not checked. Also, the precise hedonistic reason to cheat remains unknown. Besides the obvious desire to gain financial reward, competition, self-presentation, or merely the thrill of cheating may also fuel deceit.

In conclusion, as expected the dark personality traits were found to be associated with deception in a behavioural measurement, under conditions of potential for personal gain and no risk of detection. If the SD4 is to be used as a screening instrument for deception proneness, it is advisable to frame the score in likelihoods rather than in cut-off scores. Obviously, further research is needed. Meanwhile, the literature on deception detection employing other approaches than personality testing (e.g., analyses of verbal characteristic of the statement at hand, or the analysis of nonverbal behaviour of the sender) paints a pessimistic picture of accuracy rates. Hence, the use of screening measures such as the SD4 is a welcome addition that deserves future research attention. One topic of interest is whether the SD4 can predict deceitfulness under different conditions defined by the magnitude of the potential gain, the likelihood of gain, and the risk of getting caught.

## Methods

### Participants

Seven hundred and fifty-one general community volunteers participated in this study. The mean age in the sample was 28.0 years (*SD* = 11.7). There were 514 women (69%), 226 men (30%), and six persons that identified themselves as non-binary (0.8%); five participants (0.5%) did not indicate their age. Participants were recruited via university recruitment systems and through social networks of the research assistants. Participants did not receive any compensation but were instructed that the likelihood of a financial reward would increase with superior performance on the Matrix task. This was done to create an incentive to exaggerate performance, which was construed as a hedonistic lie.

### Materials

The SD4^2^ consists of 28 items, seven for each of the measured four dark traits. Items are answered on a 5-pointscale (1 = *strongly disagree*; 5 = *strongly agree*). Answers can be averaged (range: 1–5) or summed (range: 7–35 for individual traits, and 28–140 for the SD4 total score). In the current sample (*N* = 751), the mean total score for Machiavellianism was 21.74 (*SD* = 3.69; Cronbach’s *alpha* = 0.55), 19.42 (*SD* = 4.56; *α* = 0.76) for Narcissism, 14.42 (*SD* = 4.48; *α* = 0.74) for Psychopathy, 15.97 (*SD* = 5.16; *α* = 0.78) for Sadism, and 71.55 (*SD* = 12.73; *α* = 0.84) for the SD4 total score.

**Figure 2:**
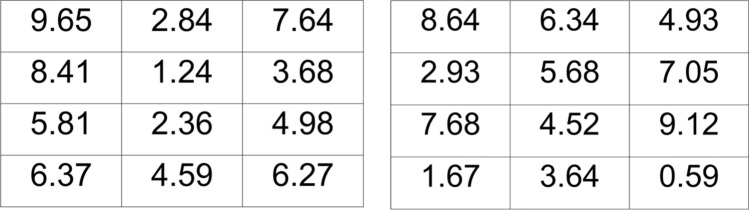
Examples of Solvable (Left) and Unsolvable (Right) Matrices. In the left panel, the matrix is solvable, because there are two numbers that add up to ten (namely 7.64 and 2.36). No such two numbers are present in the right panel which represents an unsolvable matrix. For all participants, the first thirteen matrices were solvable, and the last seven were not. Hence, participants who reported to have solved more than thirteen matrices were classified as liars.

After filling out the SD4, participants completed a puzzle task as described by Roeser et al.^9^ in which they were invited to solve as many puzzles as they could out of twenty, with a time limit of 30 s per puzzle. Figure 2 presents an example of two puzzles. In this task, the goal was to find two numbers that add up to ten. The instruction was as follows: “In this test, you will be presented with a slide with 12 numbers on it (a 4 * 3 matrix). Each number is between 0 and 10, and has two decimals. You need to figure out whether the slide contains two numbers that add up to precisely 10. In fact you will be presented with 20 matrices, one at a time. In the end, all you will be asked is how many of these twenty you think you were able to solve. Hence, you will be asked to insert a number between 0 and 20. Note that there is an increment in difficulty over the 20 trials. There is also time pressure, because each matrix will be displayed for 30 s. You may want to take notes (pen and paper) to register how many matrices you are able to solve. To increase enthusiasm, we will divide ten prices of 10 euro by lottery among the best performing participants.” Eventually, participants were asked to report how many of the twenty puzzles they had been able to solve, that is, in how many puzzles they had found two numbers adding up to ten.

Unknown to participants, the first thirteen puzzles were solvable, but the latter seven were unsolvable. Hence, participants who reported to have solved more than thirteen puzzles were classified as liars. These participants were considered to have lied to increase their chance of financial gain.

### Procedure

The project was approved by Erasmus School of Social and Behavioural Sciences’ ethics review board (ETH2122-0352). Data were collected in accordance with national regulations and guidelines. Participants were recruited via a university recruitment system and via social networks of the research assistants (convenience snowball sampling). Participants completed the study online (Qualtrics). They first gave informed consent and answered demographic questions (age and gender). Then the SD4 was completed. Finally, they completed the matrix test. The order of matrices was the same for all participants. The test started with thirteen solvable ones, after which the seven unsolvable ones followed.

## Data Availability

Data are available at https://osf.io/z83se/
